# Preparation and Chemical Properties of π-Conjugated Polymers Containing Indigo Unit in the Main Chain

**DOI:** 10.3390/ma7032030

**Published:** 2014-03-11

**Authors:** Hiroki Fukumoto, Hisashi Nakajima, Takahiro Kojima, Takakazu Yamamoto

**Affiliations:** 1 College of Biomolecular Functional Engineering, Faculty of Engineering, Ibaraki University, 4-12-1, Nakanarusawa, Hitachi 316-8511, Japan; 2 Chemical Resources Laboratory, Tokyo Institute of Technology, 4259, Nagatsuta, Midori-ku, Yokohama 226-8503, Japan; E-Mail: nkjm-hf@nifty.com; 3 Institute of Advanced Energy, Kyoto University, Gokasyo, Uji 611-0011, Japan; E-Mail: kojima@iae.kyoto-u.ac.jp

**Keywords:** π-conjugated polymer, indigo, polycondensation, H-chromophore

## Abstract

π-Conjugated polymers based on indigo unit were prepared. Dehalogenative polycondensation of *N*-hexyl-6,6′-dibromoindigo with a zerovalent nickel complex gave a homopolymer, **P(HexI)**, in 77% yield. Copolymer of *N*-hexyl-indigo and pyridine, **P(HexI-Py)**, was also prepared in 50% yield. **P(HexI)** showed good solubility in organic solvents, whereas **P(HexI-Py)** was only soluble in acids such as HCOOH. The weight-average molecular weights (*M*_w_) of **P(HexI)** and **P(HexI-Py)** were determined to be 10,000 and 40,000, respectively, by a light scattering method. Pd-catalyzed polycondensation between 6,6′-dibromoindigo with *N*-BOC (BOC = *t*-butoxycarbonyl) substituents and a diboronic compound of 9,9-dioctylfluorene afforded the corresponding alternating copolymer, **P(BOCI-Flu)**, as a deep red solid in 98% yield. **P(BOCI-Flu)** was soluble in *N*-methyl-2-pyrroridone and showed an *M*_w_ of 29,000 in GPC analysis. Treatment of **P(BOCI-Flu)** with CF_3_COOH smoothly led to a BOC-deprotection reaction to give an insoluble deep green polymer, **P(I-Flu)**, in a quantitative yield. Diffuse reflectance spectra of powdery **P(BOCI-Flu)** and **P(I-Flu)** showed peaks at about 580 nm and 630 nm, respectively, which are thought to originate from the indigo unit.

## 1. Introduction

**Chart 1.**
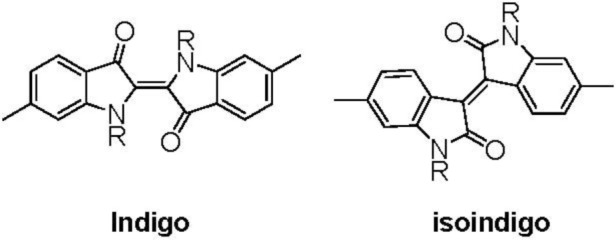
Indigo-6,6′-diyl unit and isoindigo-6,6′-diyl unit used for the component in polymer main chains (R = H, alkyl, *etc*.)

Indigo is one of the most important and popular dyes [1,2] and has widely been used at industrial level. Recently indigo [3–6] and compounds based on a isomer of indigo (*iso*indigo) [6–11] are gathering attention as active materials for electronic devices such as field effect transistors and solar cells (renaissance of color [6]); they are thought to be good candidates for electron-withdrawing (or electron-deficient) electronically active materials. Especially the isoindigo unit shown in Chart 1 has received strong attention, and various π-conjugated polymers and oligomers containing isoindigo in their main chains have been prepared [6–14] since Reynolds’ group reported synthesis of such compounds. However, examples of π-conjugated polymers containing the indigo unit in the main chain are not many [15]. Because indigo has strong historical and basic chemical backgrounds, development of the chemistry of indigo-based polymers seems to be important.

**Chart 2.**
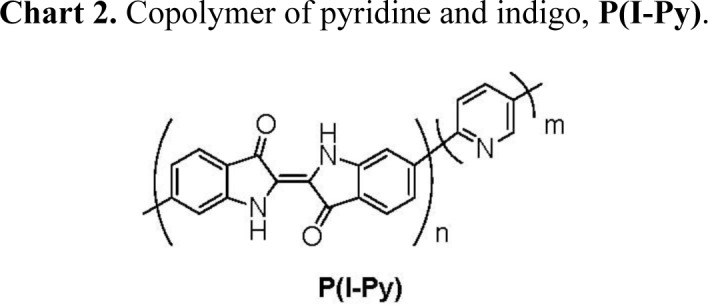
Copolymer of pyridine and indigo, **P(I-Py)**.

Previously, our group reported indigo-incorporated poly(pyridine-2,5-diyl), **P(I-Py)** shown in Chart 2, and investigated optical properties of the polymer [15,16]; e.g., film of **P(I-Py)** showed third-order nonlinear optical susceptibility, χ^(3)^, comparable to that of π-conjugated polymers with sufficiently long π-conjugation systems [15].

However, **P(I-Py)** was soluble only in special solvents such as formic acid and insoluble in common organic solvents (presumably due to the formation of intermolecular hydrogen bonds; see below), which made it difficult to investigate chemical properties of the polymer well.

**Chart 3.**
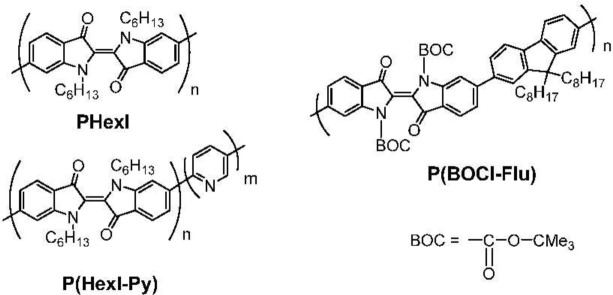
Synthesized π-conjugated indigo polymers with solubilizing and protecting groups.

As a step for getting more information about indigo-based polymers, we introduced solubilizing substituents (hexyl and BOC (*t*-butoxycarbonyl) groups) at the N–H group in the indigo unit and have prepared the following polymers shown in Chart 3. Herein we report syntheses of these polymers and UV-Vis data of the polymers.

As shown in Chart 1, the *iso*indigo-6,6′-diyl unit forms formally fully π-conjugation system along the polymer main chain. On the other hand, the indigo-6,6′-diyl unit does not have such a system and effective expansion of the π-conjugation system along the polymer main chain is not expected. However, it may be possible to form a *cross* π-conjugation system [17] via the C=O carbonyl group under certain conditions; such a cross π-conjugated unit sometimes forms expanded electron systems along the polymer chain via the unit [17–20]. In addition, when the indigo-6,6′-diyl unit (abbreviated as **In**; R = H in Chart 1) is chemically reduced, the formed **In**^2−^ might be able to form formally fully π-conjugated system along the polymer chain [16].

The BOC group is a convenient group which protects the possibly reactive N–H group of the indigo unit during the polymerization and undergoes clean thermal and acid-induced deprotection to recover the N–H group (e.g., N–COOCMe_3_ → NH + CO_2_ + CH_2_=CMe_2_) [21–26] when desired. As described below **P(BOCI-Flu)** also undergoes the clean deprotection reaction.

We now report results of the synthesis and chemical properties of the above polymers.

## 2. Results and Discussion

### 2.1. Synthesis of Polymers

#### 2.1.1. PHexI and P(HexI-Py)

**Scheme 1.**
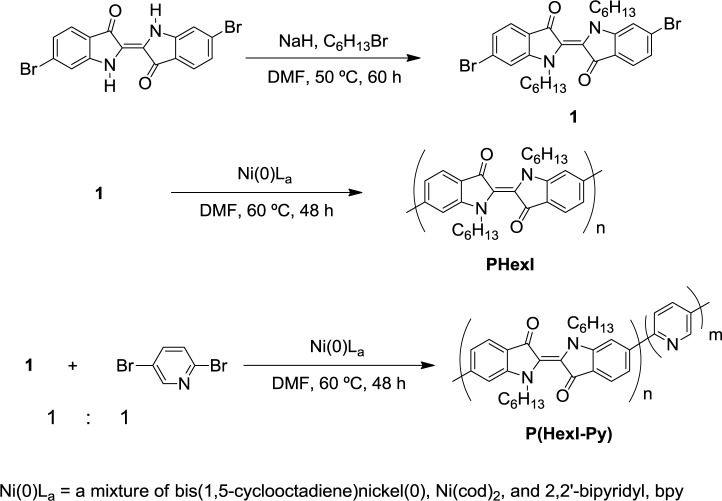
Synthetic routes to monomer **1**, **PHexI**, and **P(HexI-Py)**. The polycondensation is accompanied by the formation of NiBr_2_L_a_.

Scheme 1 shows synthetic routes for the starting monomer **1**, **PHexI** and **P(HexI-Py)**. Reaction of disodium salt of 6,6′-dibromoindigo [27] with 1-bromohexane at 50 °C gave **1** as an orange solid. Dehalogenative polycondensation [28] of **1** using zerovalent-nickel-complex ([Ni(0)L_a_]; L = neutral chelating ligands such as bpy (2,2′-bipyridyl) and cod (1,5-cyclooctadiene)) afforded **PHexI** in 77% yield. **P(HexI-Py)** was also obtained in 50% yield by a 1:1 random polycondensation of **1** with 2,5-dibromopyridine at 60 °C.

**PHexI** showed good solubility in organic solvents such as chloroform. However, **P(HexI-Py)** was only soluble in acids such as formic acid, similarly to a π-conjugated homopolymer of pyridine, poly(pyridine-2,5-diyl) [29]. **PHexI** and **P(HexI-Py)** showed weight-average molecular weights (*M*_w_) of 10,000 (in chloroform) and 40,000 (in formic acid), respectively, as measured by a light scattering method.

**Figure 1.**
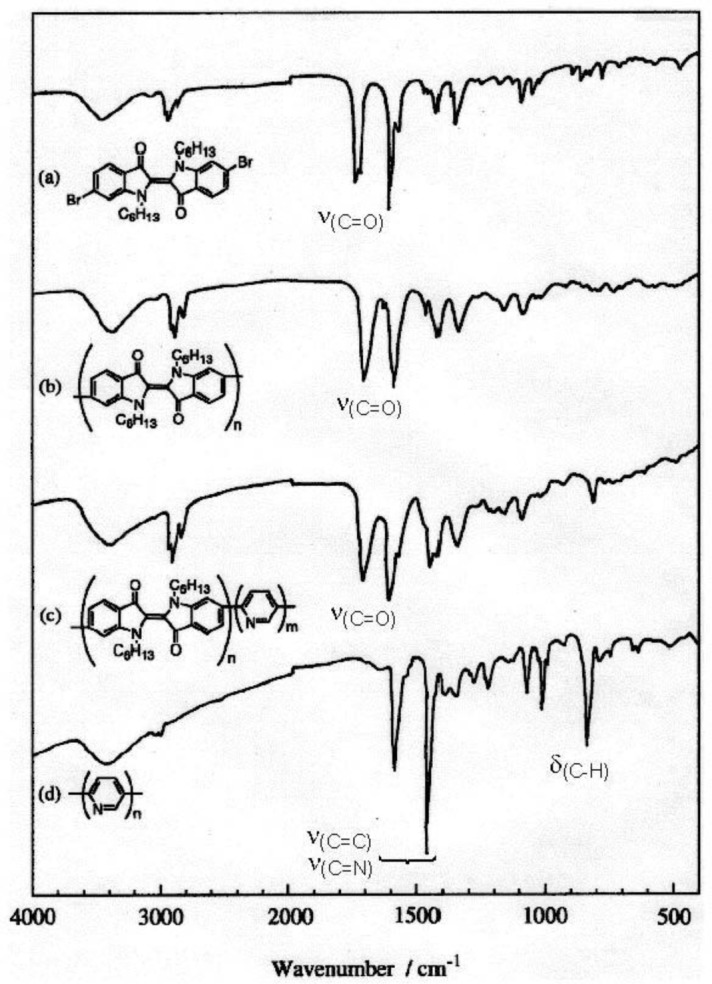
IR spectra of (**a**) **1**; (**b**) **PHexI**; (**c**) **P(HexI-Py)** and (**d**) poly(pyridine-2,5-diyl) [29].

Figure 1 shows IR spectra of **1**, **PHexI**, and **P(HexI-Py)**. As shown in Figure 1, the IR spectrum of **PHexI** resembles that of **1**, indicating that the polymerization proceeded with maintaining the unit structure of dihexylindigo. The IR spectrum of **P(HexI-Py)** shows additional absorption peaks originated from the pyridine-2,5-diyl unit at approximately 1460 and 830 cm^−1^ (*cf.* charts (c) and (d) in Figure 1), in addition to the dihexylindigo-originated IR peaks.

^1^H NMR spectra of **PHexI** and **P(HexI-Py)** are shown in Figure S1 in Supporting Materials. The area ratio between peaks in the aromatic-H region and those in the aliphatic region essentially agrees with the polymer structures. Based on the ^1^H NMR results, **P(HexI-Py)** is thought to contain the dihexylindigo and pyridine-2,5 units in agreement with the feeding ratio (1:1) of the two monomers.

#### 2.1.2. **P(BOCI-Flu)** and Its Deprotection

**Scheme 2.**
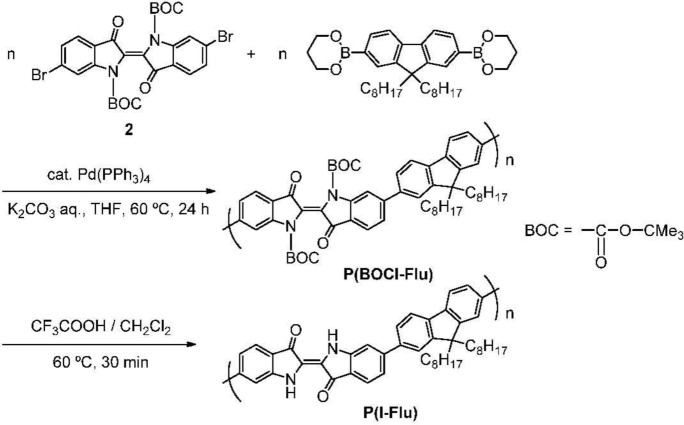
Synthetic routes to **P(BOCI-Flu)** and deprotection of the BOC group in **P(BOCI-Flu)** by treatment with acid (CF_3_COOH).

The route for the preparation of **P(BOCI-Flu)** and BOC-deprotection of **P(BOCI-Flu)** are outlined in Scheme 2. BOC-protected 6,6′-dibromoindigo (monomer **2**) was prepared according to the literature [5,30].

Pd-catalyzed polycondensation of **2** with a diboronic compound of dioctylfluorene proceeded smoothly to afford **P(BOCI-Flu)**, as a deep red solid in 98% yield. **P(BOCI-Flu)** was soluble in *N*-methyl-2-pyrrolidone (NMP) and THF, and had low solubility in chloroform. The GPC curve of this polymer (eluent = NMP) showed a unimodal molecular weight distribution, with a peak molecular weight (*M*_p_) of 18,000 and *M*_w_ of 26,000, respectively. Deprotection of the BOC group in **P(BOCI-Flu)** was carried out by treatment of this polymer with CF_3_COOH [23] and **P(I-Flu)** was obtained as a deep green solid in a quantitative yield. BOC-deprotected **P(I-Flu)** obtained thus was insoluble in common organic solvents and partly soluble in formic acid.

**Figure 2.**
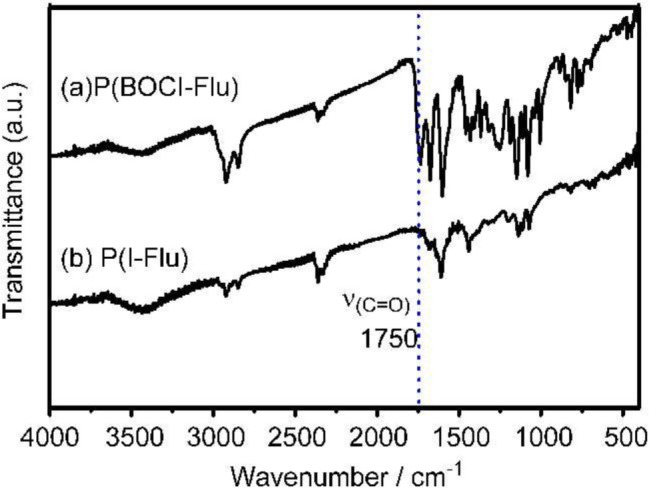
IR spectra of (**a**) **P(BOCI-Flu)** and (**b**) its deprotected (CF_3_COOH-treated) product. The absorption peak at approximately 2300 cm^−1^ is due to CO_2_ in air.

In the IR spectrum of **P(BOCI-Flu)** shown in Figure 2, the large peak observed at around 1750 cm^−1^ is assigned to ν(C=O).

**Figure 3.**
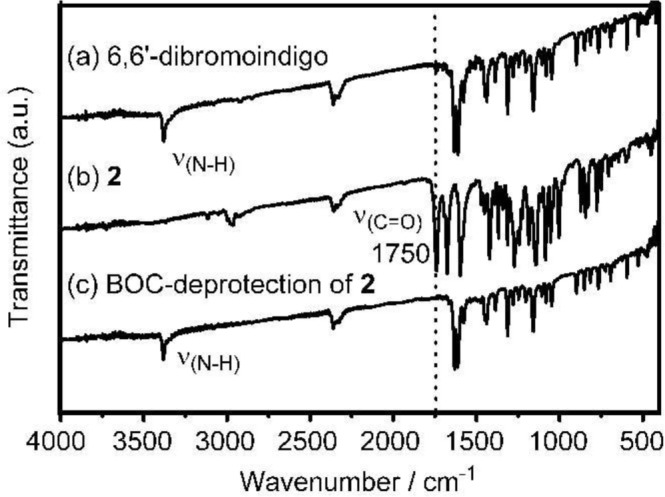
Comparison of IR spectra of (**a**) 6,6′-dibromoindigo without the BOC group; (**b**) monomer **2** with the BOC protecting groups and (**c**) BOC-deprotection (CF_3_COOH-treated) product of **2**.

The IR pattern in the C=O carbonyl region of **P(BOCI-Flu)** resembles that of monomer **2** shown in Figure 3 (curve b).

The IR spectrum of a BOC-deprotection product of monomer **2** (curve c in Figure 3) essentially agrees with that of 6,6′-dibromoindigo without the BOC group (curve a), indicating that the BOC-deprotection proceeds cleanly with the low-molecular-weight compound **2**.

The IR data shown in Figure 2 indicate that the deprotection of **P(BOCI-Flu)** with CF_3_COOH [23] also proceeds cleanly. After the deprotection of the BOC group, **P(BOCI-Flu)** became insoluble in organic solvents, in spite of the presence of two octyl groups at the fluorene unit, presumably due to the formation of intermolecular hydrogen bonds between the N–H group and the C=O carbonyl group [31,32]. Indigo and its derivatives are usually hardly soluble in common organic solvents when the *N*-H hydrogen is not substituted.

^1^H NMR spectrum of **P(BOCI-Flu)** shown in Figure S2 in Supporting Materials is reasonable for its structure. The peaks observed in the aliphatic-H region are originated from the alkyl protons of the fluorene unit and methyl protons of the BOC group in the indigo unit. The ^1^H NMR spectrum shows broad peaks in the range from δ 7.5 to 9.0 which are assigned to the aromatic-Hs.

Thermal stability of **P(BOCI-Flu)** and **P(I-Flu)** was examined by thermogravimetric analysis (TGA), and their TGA curves are shown in Figure S3 in Supporting Materials. For **P(BOCI-Flu)**, the deprotection of the BOC group started at about 175 °C, and gave a weight loss of 25%, which is roughly in agreement with the calculated weight loss in the deprotection (22.8%). **P(I-Flu)** showed 5 wt% loss temperature at 190 °C.

### 2.2. UV-Vis Absorption

**Figure 4.**
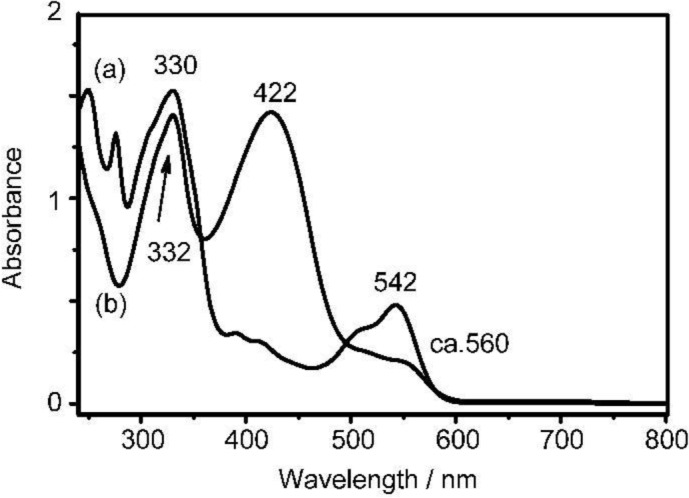
UV-Vis spectra of (**a**) monomer **2** and (**b**) **P(BOCI-Flu)** in THF.

Optical properties were mainly studied with **P(BOCI-Flu)** with good solubility in organic solvents. Figure 4 shows UV-Vis spectra of monomer **2** and **P(BOCI-Flu)** in THF. UV-Vis spectrum of **2** exhibits a peak at 542 nm, which is shifted to a shorter wavelength from that of indigo at λ_max_ = 610 nm [32]. This shift is thought to be due to a hypochromic effect of Br on indigo [31] and/or twisting of the C=C connecting bond caused by the BOC group; Miehe *et al*. [33] reported twisting of the C=C bond by introduction of methyl groups at N.

For **P(BOCI-Flu)**, the UV-Vis peak of the indigo unit becomes much weaker and a new strong peak appears at 422 nm between the peak positions of fluorene (λ_max_ = 301 nm [34]) and indigo. Both indigo (molar absorption coefficient: log (ε/M^−1^cm^−1^) = 4.3 [32]) and fluorene (log (ε/M^−1^cm^−1^) = 4.0 [34]) are strong chromophores, and the appearance of the new peak at 422 nm suggests a strong electronic interaction between the indigo and fluorene units. The formally fully π-conjugated copolymers between *iso*indigo and aromatic units also show new UV-Vis peaks [7–11], and a copolymer between isoindigo and dialkylfluorene (**P1** in reference [10]) shows a UV-Vis peak at 564 nm; the longer wavelength of **P1** suggests a better electron expansion along the polymer main chain compared with **P(BOCI-Flu)**. The UV-Vis spectrum of **P(BOCI-Flu)** film (*cf.* Figure S4 in Supporting Materials) gives an absorption pattern similar to that measured in solution, and starts at about 610 nm, which gives a band gap of 2.0 eV.

Deprotection of a dark red solid of **P(BOCI-Flu)** with CF_3_COOH (*vide ante*) gave a dark green solid **P(I-Flu)**. Because **P(I-Flu)** was not soluble, this color change was followed by diffuse reflectance spectroscopy [35] using solid samples of **P(BOCI-Flu)** and **P(I-Flu)**.

**Figure 5.**
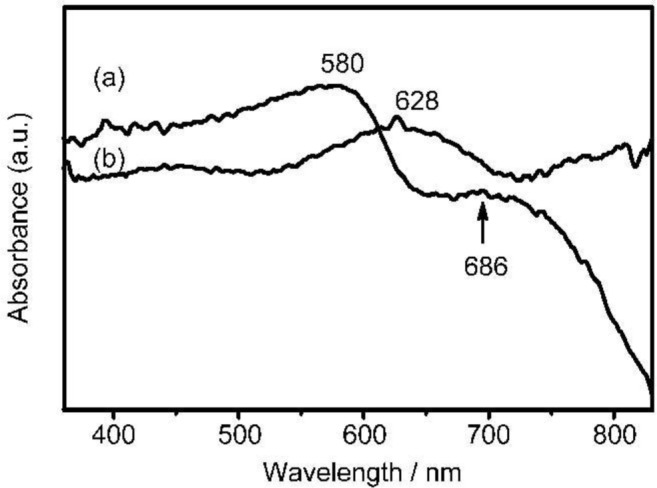
Diffuse reflectance (DR) spectra of (**a**) **P(BOCI-Flu)** and (**b**) **P(I-Flu)**.

Figure 5 shows diffuse reflectance (DR) spectra of powdery **P(BOCI-Flu)** (curve a) and **P(I-Flu)** (curve b). The DR spectrum of monomer **2** is shown in Figure S5 in Supporting Materials, the DR spectrum of **2** essentially agrees with the UV-Vis spectrum observed in THF (*cf.* Figure 4), suggesting the absence of an intermolecular electronic interaction in the solid of **2**.

The DR spectrum of **P(BOCI-Flu)** exhibits peaks at positions different from those observed with the solution sample (*cf.* Figure 4), suggesting a strong intermolecular electronic interaction between the polymer molecules in the solid state, similarly to cases of isoindigo-based polymers and oligomers; the UV-Vis spectra of solid samples of the isoindigo-based polymers and oligomers sometimes showed a bathochromic shift from those of solutions [7,10].

By the deprotection, the absorption bands seem to be shifted to longer wavelengths, and one of the absorption bands seems to shift to a near infrared region. This shift accompanied with the deprotection is associated with a UV-Vis peak shift of *N*,*N*-diBOC-indigo to indigo, which is induced by acid [36].

UV-Vis spectra of **P(HexI)** and **P(HexI-Py)** in HCOOH are shown in Figure S6 in Supporting Materials. They show absorption peaks at 300 nm (**P(HexI)**) and 330 nm (**P(HexI-Py)**), respectively, which are thought to originate from the indigo unit. However, the peak at around 600 nm, which is characteristic of indigos and was observed in the spectrum of **P(BOCI-Flu)**, was not detected, suggesting that electronic state of the H-chromophore in the indigo unit is affected by the interaction between proton(s) of HCOOH and the carbonyl group in the indigo unit.

### 2.3. Electrochemical Behavior

Electrochemical responses of **P(HexI-Py)** and **P(BOCI-Flu)** were investigated by cyclic voltammetry (CV) in CH_3_CN. Film of **P(HexI-Py)** cast on a Pt plate received electrochemical reduction, reflecting an electron deficient nature of the pyridine ring, and a CV reduction peak appeared at −2.2 V *vs*. Ag^+^/Ag (*cf.* Figure S7 in Supporting Materials). The peak position is located near that observed for polypyridines [29]. In the case of **P(BOCI-Flu)**, it became soluble in the CH_3_CN solution when reduction potential over −2.0 V Ag^+^/Ag was applied, suggesting the formation of an ionic polymer. Because of this, the investigation of CV of **P(BOCI-Flu)** was difficult.

## 3. Experimental Section

### 3.1. Measurements and Procedure

NMR spectra were recorded on JEOL JNM-EX90 and EX400 spectrometers (JEOL Ltd., Akishima, Japan). IR spectra were taken using JASCO-IR 810 and 460 spectrometers (JASCO Corporation, Hachioji, Japan). UV-vis spectra were measured with Shimadzu UV-3100PC and UV-2500PC spectrometers (SHIMADZU Corporation, Kyoto, Japan). Gel permeation chromatography (GPC) of **P(BOCI-Flu)** was performed at TOSOH Analysis and Research Center Co., Ltd using a TOSOH HLC-8120GPC liquid chromatograph (Tosoh Corporation, Tokyo, Japan), NMP as the eluent, and polystyrene standards. Measurements of *M*_w_ by the light scattering method were carried out using an OTSUKA DLS-700 spectrophotometer (Otsuka Electronics Co., Ltd., Hirakata, Japan). Diffuse reflectance spectra of powdery polymers and monomer **2** were measured on a Shimadzu UV-3101PC spectrometer equipped with an integrating sphere assembly and converted to UV-Vis absorption spectra using Kubelka-Munk theory [35]. BaSO_4_ was the reference standard. TGA curves were taken using Rigaku TG 8120 thermometric system (Rigaku Corporation, Tokyo, Japan). Cyclic voltammetry (CV) was performed with a Hokuto Denko HA-301 galvanostat/potentiostat (HOKUTO DENKO Corporation, Tokyo, Japan) and a Hokuto Denko HB-104 function generator. Cyclic voltammograms were obtained for films of the polymers laid on a Pt plate (1 cm × 1 cm) in a CH_3_CN solution of [NEt_4_][BF_4_] (0.1 M) with a Pt counter electrode and an Ag^+^/Ag reference electrode. The preparation of polymers was carried out under inert gas using standard Schlenk techniques.

### 3.2. Materials

6,6′-Dibromoindigo [27], BOC-protected 6,6′-dibromoindigo (**2**) [5,30], Ni(cod)_2_ (cod = 1,5-cyclooctadiene) [37,38] and Pd(PPh_3_)_4_ [39] were prepared as previously reported. Commercially available Ni(cod)_2_ was also used. 9,9-Dioctylfluorene-2,7-diboronic acid bis(1,3-propanediol) ester was purchased from Sigma-Aldrich Co. LLC (Sigma-Aldrich Co. LLC., St. Louis, USA).

### 3.3. Synthesis of N,N′-dihexyl-6,6′-dibromoindigo (**1**)

To a dispersion of NaH (60% in paraffin oil) (0.24 g, 6.0 mmol) in DMSO (60 mL) was added 6,6′-dibromoindigo (1.0 g, 2.4 mmol) at 50 °C, and the mixture was stirred overnight at 50 °C for 12 h. 1-Bromohexane (2.4 g, 14.4 mmol) was added to the mixture, and the mixture was stirred at 65 °C for 48 h. After cooling to room temperature (rt), the reaction mixture was extracted with chloroform. The extract was washed with water and dried over MgSO_4_. Solvents were removed by evaporation, and the product was purified by column chromatography on silica (eluent: hexane/ethyl acetate = 5/1) and recrystallization using hexane to afford **1** (0.13 g, 9%) as orange crystals. ^1^H NMR (90 MHz, CDCl_3_): δ = 7.46 (d, 2H, *J* = 7.9 Hz), 7.27 (dd, 2H, *J* = 7.9 and 1.4 Hz), 7.07 (d, 2H, *J* = 1.4 Hz), 3.70 (t, 4H, *J* = 7.1 Hz), 1.69 (m, 4H), 1.35 (br, 12H), 0.89 (br, 6H). IR (KBr pellet, cm^−1^): ν = 2954, 2928, 2858, 1732, 1602, 1428, 1355, 1100, 1058, 906, 869, 787.

### 3.4. Synthesis of **PHexI**

To a mixture of Ni(cod)_2_ (0.18 g, 0.65 mmol), 2,2′-bipyridine (0.65 mmol) and DMF (50 mL) was added **1** (0.294 g, 0.50 mmol) at 60 °C, and the mixture was stirred at 60 °C for 48 h. After cooling, the reaction mixture was poured into an aqueous ammonium solution to give a precipitate. The precipitate was collected by filtration and washed with aqueous ammonia, diluted HCl_aq_, aqueous ammonia containing EDTA (ethylenediaminetetraacetic acid), aqueous ammonia, and methanol in this order. The solid was dried under reduced pressure to afford **PHexI** (0.166 g, 77% yield) as a yellowish brown powder. ^1^H NMR (90 MHz, CDCl_3_): δ = 7.50–6.63 (6H), 3.67 (4H), 1.69 (4H), 1.33 (12H), 0.91 (6H). IR (KBr pellets, cm^−1^): ν = 3392, 2892, 1703, 1586, 1415, 1334, 1083. Light scattering analysis (in formic acid): *M*_w_ = 10,000.

### 3.5. Synthesis of **P(HexI-Py)**

A mixture of 2,5-dibromopyridine (0.118 g, 0.50 mmol) and **1** (0.294 g, 0.50 mmol) was added to a DMF (30 mL) suspension of Ni(cod)_2_ (0.36 g, 1.30 mmol), 2,2′-bipyridine (1.30 mmol) at 60 °C, and the mixture was stirred at 60 °C for 48 h. Work-up of the reaction mixture was carried out in a manner similar to that for **PHexI**. After work-up, 0.126 g of **P(HexI-Py)** was yielded as a green powder. 50% yield. ^1^H NMR (90 MHz, CF_3_COOD): δ = 9.83 (1H), 9.14 (1H), 8.81 (1H), 8.1–6.8 (6H), 3.94 (4H), 1.92 (4H), 1.50 (12H), 0.94 (6H). IR (KBr pellet, cm^−1^): ν = 3392, 2928, 1712, 1604, 1442, 1334, 1083, 813. Light scattering (in chloroform): *M*_w_ = 40,000.

### 3.6. Synthesis of **P(BOCI-Flu)**

A mixture of **2** (0.46 g, 0.74 mmol), 9,9-dioctylfluorene-2,7-diboronic acid bis(1,3-propanediol) ester (0.40 mg, 0.71 mmol), distilled water (25 mL), K_2_CO_3_ (1.5 g) and Pd(PPh_3_)_4_ (55 mg, 0.05 mmol) and THF (25 mL) was stirred at 60 °C for 24 h. The reaction mixture was cooled to rt, and the organic layer was poured into methanol to obtain a dark red solid, which was collected by filtration and dissolved in NMP. The NMP solution was poured into methanol. The dark red precipitate was separated by filtration, washed with methanol repeatedly, and dried under reduced pressure to yield a dark red solid of **P(BOCI-Flu)** (0.61 g, 98% yield). ^1^H NMR (400 MHz, THF-*d*_8_): δ = 8.49 (2H), 7.84 (10H), 2.48 (18H), 1.62 (12H), 1.13 (16H), 0.80 (6H). IR (KBr pellet, cm^−1^): ν = 3500, 2924, 2848, 1750, 1676, 1600, 1431, 1251, 1147, 1080, 1004, 817. GPC (eluent = NMP, *vs*. polystyrene standards): *M*_n_ = 3,000. *M*_w_ = 26,000.

### 3.7. Boc-Deprotection of **P(BOCI-Flu)**: Treatment with CF_3_COOH

CF_3_COOH (*ca.* 2 mL) was added to a suspension of the **P(BOCI-Flu)** (20 mg) in dichloromethane (*ca.* 2 mL) at rt. The mixture was stirred for 30 min at 60 °C and poured into 10 mL of water. After the removal of dichloromethane by evaporation at 60 °C, K_2_CO_3_ was added to neutralize the solution. The supernatant solution was decanted, and the precipitate was washed with methanol (20 mL). The powder was separated by centrifugation and dried under reduced pressure. **P(I-Flu)** (15 mg, quantitative yield. IR (KBr pellets, cm^−1^): ν = 3398, 2924, 2850, 1608, 1440, 1355, 1138.

## 4. Conclusions and Scope

Soluble **P(HexI)**, **P(HexI-Py)**, and **P(BOCI-Flu)** with the alkyl and BOC groups have been prepared by organometallic polycondensation, and **P(BOCI-Flu)** underwent a clean deprotection reaction. The obtained **P(I-Flu)** by the deprotection reaction is expected to give basic chemical information about polymers consisting of the non-substituted indigo unit in the polymer main chain. UV-Vis data of **P(BOCI-Flu)** suggested electronic interaction between the indigo and fluorene units, and the electronic interaction seems to give a new electronic state along the polymer chain. The DR spectrum of the deprotected **P(I-Flu)** shows a bathochromic shift from that of **P(I-Flu)** and suggests intermolecular electronic interaction between **P(I-Flu)** molecules in the solid state. Synthesis of various polymers using monomers **1** and **2** is expected to give a new class of polymers (as isomeric polymers of the isoindigo-based polymers) which show interesting chemical properties.

## Supplementary Information

Contains additional data on ^1^H NMR spectra of **PHexI**, **P(HexI-Py)**, and **P(BOCI-Flu)**, TGA curves of **P(BOCI-Flu)** and **P(I-Flu)**, UV-Vis spectra of **P(HexI-Py)** and **P(HexI)** in HCOOH, and **P(BOCI-Flu)** film, diffuse reflectance spectrum of monomer **2**, and CV chart of **P(HexI-Py)** film.

## References

[1] McKinley C.E. (2011). Indigo in Search of the Color That Seduced the World.

[2] Balfour-Paul J. (2006). Indigo.

[3] Irimia-Vladu M., Głowacki E.D., Troshin P.A., Schwabegger G., Leonat L., Susanova D.K., Krystal O., Ullah M., Kanbur Y., Bodea M. (2012). Indigo—A natural pigment for high performance ambipolar organic field effect transistors and circuits. Adv. Mater.

[4] Kojima H., Mori T. (2012). Estimated mobility of ambipolar organic semiconductors, indigo and diketopyrrolopyrrole. Chem. Lett.

[5] Głowacki E.D., Voss G., Demirak K., Havlicek M., Sünger N., Okur A.C., Monkowius U., Gasiorowski J., Leonat L., Sariciftci N.S. (2013). A facile protection-deprotection route for obtaining indigo pigments as thin films and their applications in organic bulk heterojunctions. Chem. Commun.

[6] Robb M.J., Ku S.-Y., Brunetti F.G., Hawker G.J. (2013). A renaissance of color: New structures and building blocks for organic electronics. J. Polym. Sci. Part A Polym. Chem.

[7] Mei J., Graham K.R., Stalder R., Reynolds J.R. (2010). Synthesis of Isoindigo-based oligothiophenes for molecular bulk heterojunction solar cells. Org. Lett.

[8] Stalder R., Mei J., Subbiah J., Grand C., Estrada L.A., So F., Reynolds J.R. (2011). n-Type conjugated polyisoindigos. Macromolecules.

[9] Stalder R., Mei J., Subbiah J., Grand C., Estrada L.A., So F., Reynolds J.R. (2012). An isoindigo and dithieno[3,2-b:2′,3′-d]silole copolymer for polymer solar cell. Polym. Chem.

[10] Stalder R., Mei J., Reynolds J.R. (2010). Isoindigo-based donor-acceptor conjugated polymers. Macromolecules.

[11] Estrada L.A., Liu D.Y., Salazar D.H., Dyer A.L., Reynolds J.R. (2012). Poly[bis-EDOT-isoindigo]: An electroactive polymer applied to electrochemical supercapacitors. Macromolecules.

[12] Ashraf R.S., Kronemeijer A.J., James D.I., Sirringhaus H., McCulloch I. (2012). A new thiophene substituted isoindigo based copolymer for high performance ambipolar transistors. Chem. Commun.

[13] Grenier F., Berrouard P., Poulit J.-R., Tseng H.-R., Heeger A.J., Leclerc M. (2013). n-Type isoindigo copolymers. Polym. Chem.

[14] Sonar P., Tan H.-S., Sun S., Lam Y.M., Dodabalapur A. (2013). Isoindigo dye incorporated copolymers with naphthalene and anthracene: Promising materials for stable organic field effect transistors. Polym. Chem.

[15] Yamamoto T., Kizu K., Maruyama T., Ooba N., Tomaru S., Kubota K. (1994). Poly(pyridine-2,5-diyl) containing indigo unit and optical third harmonic generation from its film. Chem. Lett.

[16] Yamamoto T., Kizu K. (1995). Copolymer of pyridine and indigo. A π-conjugated polymer with color center. J. Phys. Chem.

[17] Gholami M., Tykwinski R.R. (2006). Oligomeric and polymeric systems with cross-conjugated π-framework. Chem. Rev.

[18] Streitwieser A. (1961). Macromolecular Orbital Theory for Organic Chemistry.

[19] Fang Q., Yamamoto T. (2003). New soluble unsaturated polyketone derived from diaryldenecycloalketone: Synthesis and optical and electrochemical properties of π-conjugated poly(diaryldenecyclohexanone) with long side chains. Polymer.

[20] Abe M., Yamamoto T. (2013). New cross π-conjugated polyketones: Synthesis and chemical properties. Synth. Met.

[21] Zhang X.-X., Sadighi J.P., Mackewitz T.W., Buchwald S.L. (2000). Efficient synthesis of well-defined, high molecular weight polyanilines under mild conditions via palladium-catalyzed amination. J. Am. Chem. Soc.

[22] Sadighi J.P., Singer R.A., Buchwald S.L. (1998). Palladium-catalyzed synthesis of monodisperse, controlled-length, and functionalized oligoanilines. J. Am. Chem. Soc.

[23] Srivasan N., Yurek-George A., Ganesan A. (2005). Rapid deprotection of *N*-Boc by TFA combined with freebase generation using basic ion-exchanging resins. Mol. Divers.

[24] Yamamoto T., Yoshizawa M., Mahmut A., Abe M., Kuroda S.-I., Imase T., Sasaki S. (2005). Preparation of new π-conjugated polypyrroles by organometallic polycondensations. Synthesis of *N*-BOC (*t*-butoxycarbonyl) and *N*-phenylethynyl polymers, thermal deprotection of the BOC group, and packing structure of the *N*-phenylethynyl polymer. J. Polym. Sci. Part A Polym. Chem.

[25] Horie M., Yamaguchi I., Yamamoto T. (2006). Synthesis of new poly(arylamine)s (aryl = oligo-*p*-phenyl or pyridyl) by organometallic polycondensation and chemical properties of the polymers. Macromolecules.

[26] Koie S., Tanaka G., Fukumoto H., Koizumi T., Yamamoto T. (2013). Preparation of π-conjugated polymers consisting of 1-aminopyrrole and 4-amino-1,2,4-triazole units. React. Func. Polym.

[27] Imming P., Imhof I., Zentgraf M. (2001). An improved synthetic procedure for 6,6′-dibromo indigo (tyrian purple). Synth. Commun.

[28] Yamamoto T. (2010). Synthesis of π-conjugated polymers by organometallic polycondensation. Bull. Chem. Soc. Jpn.

[29] Yamamoto T., Maruyama T., Zhou Z.-H., Ito T., Fukuda T., Yoneda Y., Begum F., Ikeda T., Sasaki S. (1994). π-conjugated poly(pyridine-2,5-diyl), poly(2,2′-bipyridine-5,5′-diyl), and their alkyl derivatives. Preparation, linear structure, function as a ligand to form their transition metal complexes, catalytic reactions, n-type electrically conducting properties, optical properties, and alignment on substrates. J. Am. Chem. Soc.

[30] Ichimura K., Arimitsu K., Tahara M. (2004). Photoacid-catalysed pigmentation of dyestuff precursors enhanced by acid amplifiers in polymer films. J. Mater. Chem.

[31] Jacqemin D., Preat J., Wathlet V., Perpete E.A. (2006). Substitution and chemical environmental effects on the absorption spectrum of indigo. J. Chem. Phys.

[32] Seixas de Melo J., Moura A.P., Melo M.J. (2004). Photophysical and spectroscopic studies of indigo derivatives in their keto and leuco forms. J. Phys. Chem. A.

[33] Miehe G., Süsse P., Kupcik V., Egert E., Nieger M., Kunz G., Gerke R., Knieriem B., Niemeyer M., Lüttke W. (1991). Light absorption as well as crystal and molecular structures of *N,N*-dimethylindigo: An example of use of synchrotron radiation. Angew. Chem. Int. Ed. Engl.

[34] Weast R.C. CRC Handbook of Chemistry and Physics.

[35] Kubelka P., Munk F. (1931). Ein beitrag zur optik der farbanstriche. Z. Tech. Phys.

[36] Tahara M., Arimitsu K., Park S.-W., Lee S., Ichimura K. (2000). Monitoring the acidolytic behavior of a latent pigment enhanced by an acid amplifier in polymer films. J. Photopolym. Sci. Technol.

[37] Wilke G. (1988). Contribution to organonickel chemistry. Angew. Chem. Int. Ed.

[38] Schunn R.A., Ittel S.D., Cushing M.A., Baker R., Gilbert R.J., Madden D.P. (1990). Bis(1,5-cyclooctadiene)nickel(0). Inorg. Synth.

[39] Coulson D.R. (1972). Tetrakis(triphenylphosphine)palladium(0). Inorg. Synth.

