# Supplementary material for: Preparation and Chemical Properties of π-Conjugated Polymers Containing Indigo Unit in the Main Chain
Source: Materials (Basel). 2014 Mar 11;7(3):2030–43. doi: 10.3390/ma7032030 (PMC5453285; doi:10.3390/ma7032030)

Supplementary Information

Figure S1. ^{1}H NMR spectra of (**a**) **PHexI** in $CDCl_3$ and (**b**) **P(HexI-Py)** in CF_3COOH.

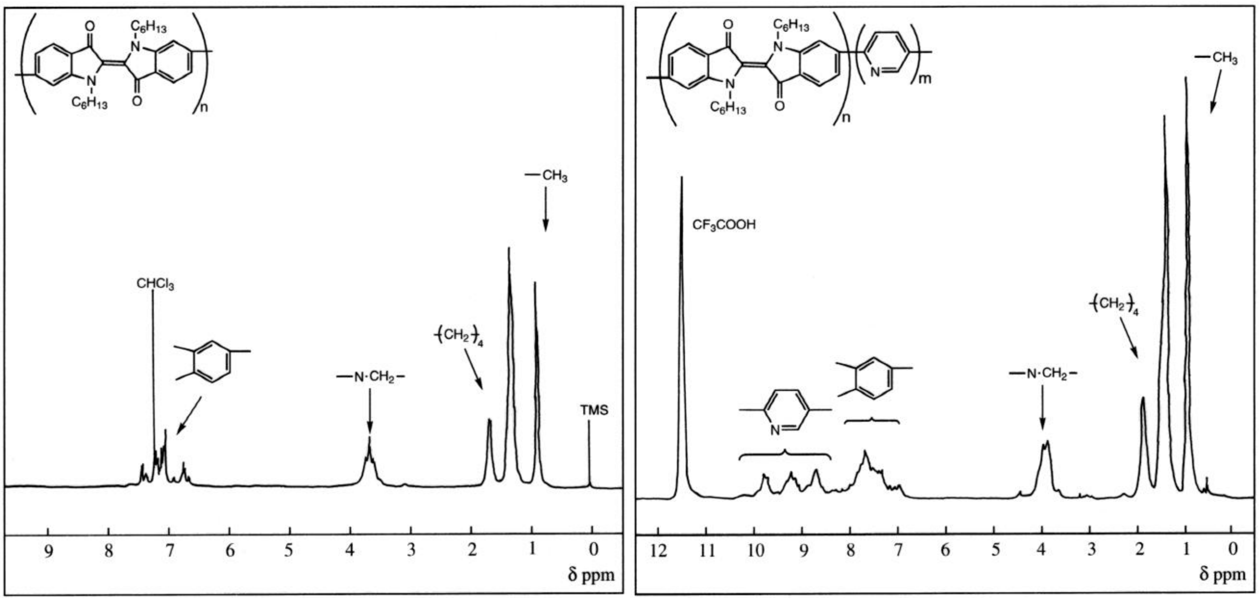

(**a**) (**b**)

Figure S2. ^{1}H NMR spectrum of **P(BOCI-Flu)** in THF-d_8. Peaks wit asterisk (*) are due to NMP used for reprecipitation of **P(BOCI-Flu)**.

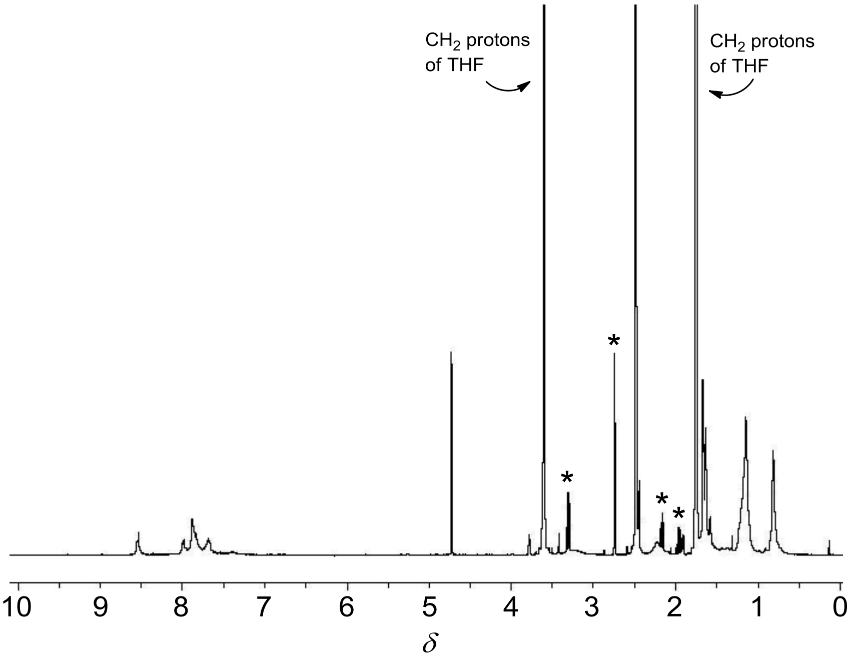

Figure S3. TG curves of **P(BOCI-Flu)** and **P(I-Flu)**.

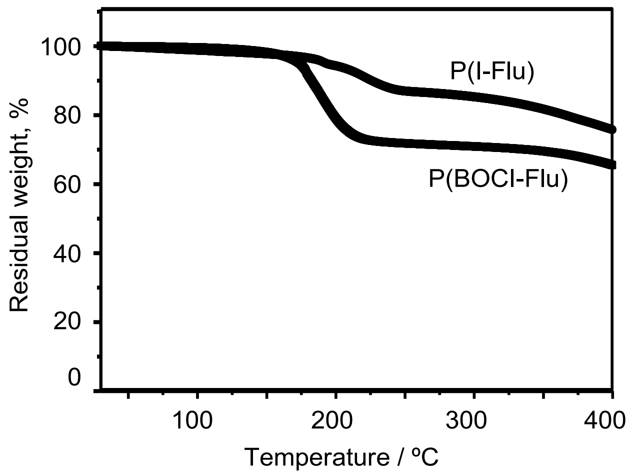

Figure S4. UV-Vis spectrum of **P(BOCI-Flu)** film cast on a quartz glass plate.

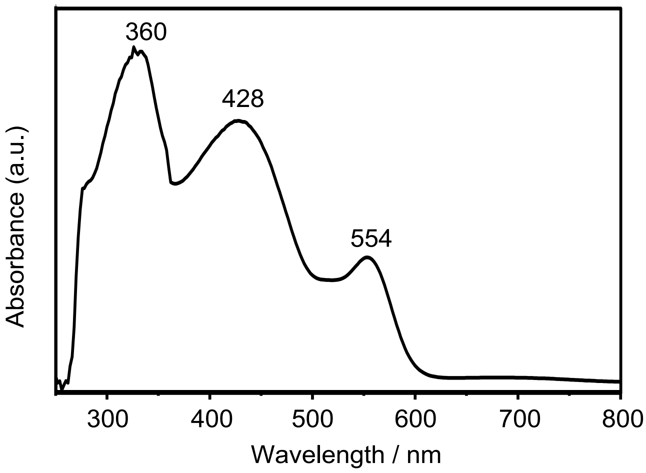

Figure S5. Diffuse reflectance (DR) spectrum of monomer **2**.

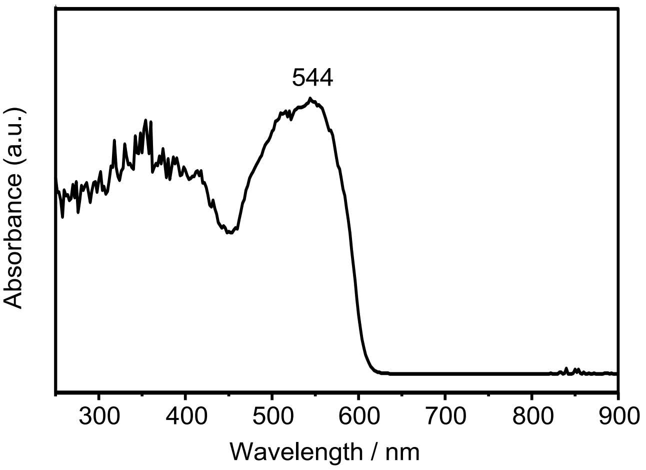

Figure S6. UV-Vis spectra of **P(HexI-Py)** and **P(HexI)** in HCOOH.

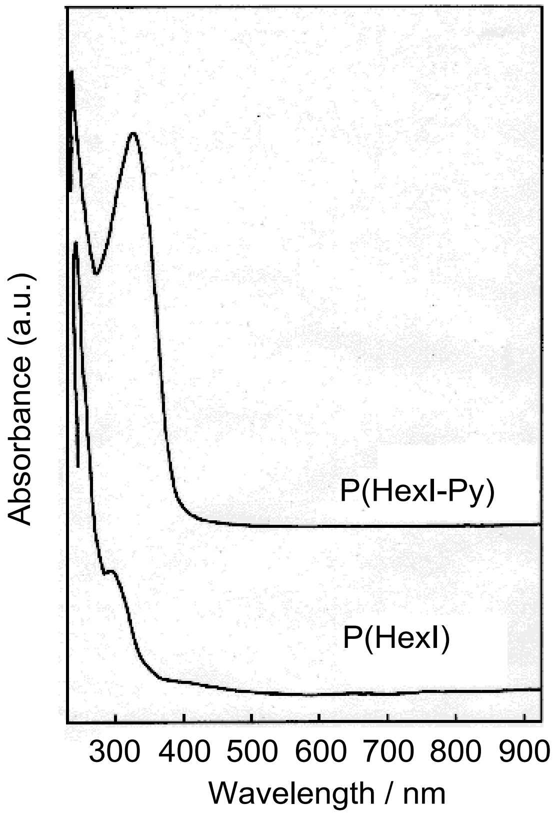

Figure S7. Cyclic voltammogram of **P(HexI-Py)** film cast on a Pt (1 cm × 1 cm) electrode in an acetonitrile solution containing 0.1 M [NEt_4] [BF_4]. Sweep rate is 20 mV/s.

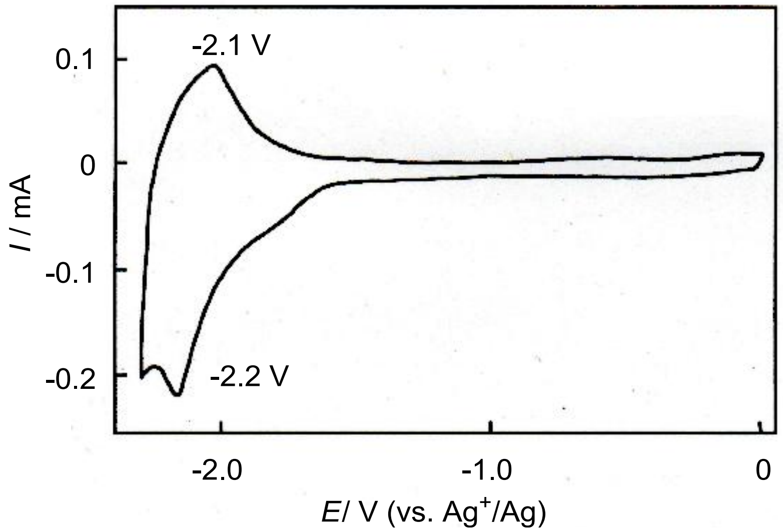

Supplement: Supplementary file 1 [file materials-07-02030-s001.pdf]
